# Long-acting lenacapavir acts as an effective preexposure prophylaxis in a rectal SHIV challenge macaque model

**DOI:** 10.1172/JCI167818

**Published:** 2023-08-15

**Authors:** Elena Bekerman, Stephen R. Yant, Laurie VanderVeen, Derek Hansen, Bing Lu, William Rowe, Kelly Wang, Christian Callebaut

**Affiliations:** Gilead Sciences Inc., Foster City, California, USA.

**Keywords:** AIDS/HIV, Drug therapy, Pharmacology

## Abstract

Long-acting antiretroviral agents for preexposure prophylaxis (PrEP) represent a promising new alternative to daily oral regimens for HIV prevention. Lenacapavir (LEN) is a first-in-class long-acting capsid inhibitor approved for the treatment of HIV-1 infection. Here, we assessed the efficacy of LEN for PrEP using a single high-dose simian-human immunodeficiency virus (SHIV) rectal challenge macaque model. In vitro, LEN showed potent antiviral activity against SHIV, as it did for HIV-1. In macaques, a single subcutaneous administration of LEN demonstrated dose proportional increases in and durability of drug plasma levels. A high-dose SHIV inoculum for the PrEP efficacy evaluation was identified via virus titration in untreated macaques. LEN-treated macaques were challenged with high-dose SHIV 7 weeks after drug administration, and the majority remained protected from infection, as confirmed by plasma PCR, cell-associated proviral DNA, and serology testing. Complete protection and superiority to the untreated group was observed among animals whose LEN plasma exposure exceeded its model-adjusted clinical efficacy target at the time of challenge. All infected animals had subprotective LEN concentrations and showed no emergent resistance. These data demonstrate effective SHIV prophylaxis in a stringent macaque model at clinically relevant LEN exposures and support the clinical evaluation of LEN for HIV PrEP in humans.

## Introduction

HIV/AIDS continues to pose a major public health threat globally, particularly in low- and middle-income countries. In the absence of a curative therapy or an effective prophylactic vaccine, other modes of HIV infection prevention are critical for combating the HIV/AIDS epidemic. Daily oral preexposure prophylaxis (PrEP) has proven highly effective at preventing new infections when taken as instructed. However, of 1.2 million people with PrEP indications in the US, less than 25% use PrEP as of 2020, and coverage remains uneven across racial and ethnic groups, age groups, and for transgender individuals (1). Low uptake is driven, in part, by the challenges associated with medication adherence crucial for the prophylactic effect of the daily oral PrEP, social stigma, and health system demands. Less frequent dosing alternatives can help address the unmet medical need among those not benefiting from currently available PrEP options (2). Long-acting PrEP agents may increase convenience, lower stigma associated with daily pill taking, reduce clinical visits, and hence, increase adherence and outcomes.

HIV capsid inhibitors represent a novel class of highly potent antivirals with biophysical and biochemical properties that make them amenable to long-acting formulation (3). Lenacapavir (LEN) is a potent and selective multistage inhibitor of HIV-1 capsid function, with demonstrated safety and antiviral efficacy following twice-yearly subcutaneous dosing in people living with HIV (4). Based on these data, LEN has been approved for the treatment of multidrug-resistant HIV-1 infection in combination with other antiretroviral(s) in multiple countries.

Macaque models for PrEP have been used to evaluate the efficacy of antiretroviral agents against various modes of virus transmission for over 2 decades (5–8). These models have been used to establish drug correlates of protection, define prophylactic windows of protection after exposure, and inform the design of clinical PrEP trials. Clinical trial data have, in turn, validated the high predictive value of the macaque models for PrEP (9). We have previously reported that GS-CA1, a structural analog of LEN exhibiting the same capsid-dependent multistage mechanism of action, provided significant protection against new SHIV infection in a repeat rectal challenge macaque model (10). The use of GS-CA1, which has a plasma half-life in macaques that is shorter than that of LEN, allowed us to efficiently examine protection against repeat challenges across a wide range of drug exposures. However, a limitation of the repeat SHIV challenge model is its reliance on an assumption regarding the infection-to-detection lag time, generally 1–2 weeks, to estimate protective drug levels. A long-acting antiretroviral drug can suppress viral replication, resulting in a diagnostic delay of a new infection and, hence, an inability to establish drug exposure at the time of seeding of an infection. To overcome this limitation in the current study, we set out to characterize LEN’s pharmacokinetic profile in rhesus macaques and evaluate its prophylactic efficacy at clinically relevant drug exposures using a high-dose single rectal challenge model. We confirmed that, like GS-CA1, LEN offered significant protection against SHIV challenge in macaques. After mathematically adjusting for the lower potency of LEN against SHIV in macaques compared with HIV-1 in humans, we observed complete protection against infection at drug exposures at or above the minimum clinical target achievable with twice-yearly subcutaneous injectable LEN.

## Results

### In vivo SHIV titration to select high challenge dose.

**Figure 1:**
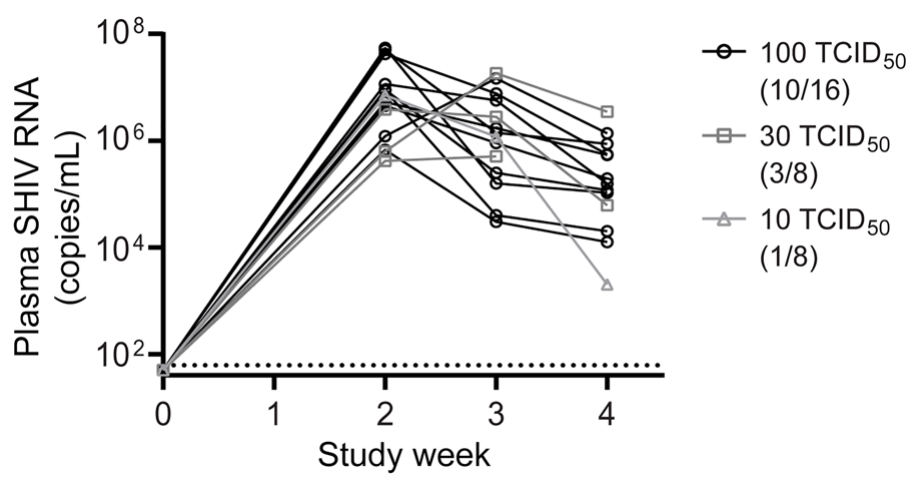
Early infection viral load kinetics among untreated animals during virus titration study. Plasma SHIV viral loads measured by gag RT-qPCR in untreated rhesus macaques that became infected following a single rectal challenge with the specified inoculum of SHIV.SF162P3. The number of infections observed per total challenges performed with a given inoculum is noted in parentheses. Plasma viral RNA monitoring was performed through week 4 after challenge, at which time viremic animals were placed on combination antiretroviral therapy. Each symbol represents an average of 3 technical assay replicates. Assay limit of detection is 62 copies per mL.

While a relatively low virus inoculum paired with multiple transmission events provides a physiologically relevant preclinical model to evaluate PrEP efficacy, the determination of actual drug exposure at the time of infection is confounded by the inability to precisely establish the timing of infection using existing analytical tools. To circumvent this challenge while evaluating the PrEP efficacy of LEN, we opted to perform a single “high-dose” challenge study. To this end, a SHIV.SF162.P3 stock expanded in activated rhesus PBMCs (50% tissue culture infectious dose [TCID_50_] = 1.024 × 10^4^/mL) was titrated in vivo over 5 rounds of increasing rectal mucosal exposure. Eight rhesus macaques per round were challenged with titers ranging from 0.625 to 100 TCID_50_ in order to select a “high” dose, yielding at least 50% infection rate per challenge (Table 1). Plasma viral loads were quantified weekly to monitor the infection status and calculate infectivity at each inoculum level (Figure 1). Peak viremia was reached by week 2 or 3 after challenge and began to decline by week 4, at which point all animals confirmed as SHIV positive were placed on daily antiretroviral therapy (ART) to prevent disease progression.

An initial inoculum of 0.625 TCID_50_ yielded no infections; however, all subsequent higher doses resulted in an increasing fraction of animals becoming viremic (Table 1 and Figure 1). After achieving 50% animal infection rate (4 of 8 infected) on the first round of challenge with 100 TCID_50_, this inoculum was retested in another group of 8 animals to increase the confidence in the virus dose selection for the efficacy study. The latter resulted in 6 of 8 animals becoming infected (i.e., 75% infection rate). At the conclusion of the titration, 14 new infections were recorded of a total of 40 challenges and displayed similar kinetics of replication, with peak viremia ranging between 4 × 10^5^ and 5 × 10^7^ SHIV copies/mL across study groups (Figure 1). The logistic regression model was then applied to compute the half-maximal animal infectious dose (AID_50_), equal to 77 TCID_50_, as well as the animal infection rate corresponding to the 100 TCID_50_ inoculum, 65.3% infection (95% CI, 40.3–84.0) (Table 1 and Supplemental Figure 1; supplemental material available online with this article; https://doi.org/10.1172/JCI167818DS1).

Because the infection rate with 100 TCID_50_ inoculum exceeded the 50% infection target, it was selected for the single high-dose challenge efficacy study. The 16 animals inoculated with 100 TCID_50_ during the titration served as controls for estimating LEN PrEP efficacy.

### Antiviral activity and pharmacokinetic profile of LEN in macaques.

**Figure 2:**
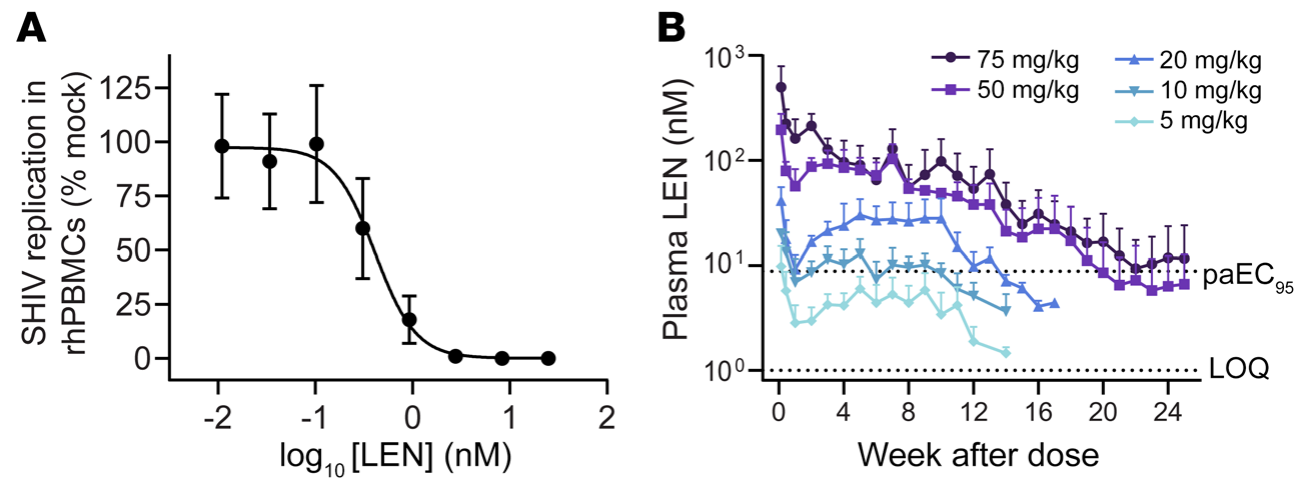
LEN antiviral activity in vitro and long-acting pharmacokinetics profile in rhesus macaques. (**A**) Representative antiviral dose–response curve for LEN in rhesus PBMCs (rhPBMCs) acutely infected with SHIV-SF162P3. Data are shown as the mean ± SD values from 1 of 7 assays (*n* = 3 biological replicates each). (**B**) Plasma LEN levels measured by mass spectrometry following a single subcutaneous administration at specified dose levels (*n* = 4/group). Data are shown as the mean ± SD. values. The higher dotted line represents the rhesus PBMC paEC_95_ for LEN (8.8 nM). The lower dashed line represents the assay limit of quantification (LOQ, 1 nM).

The in vitro antiviral potency of LEN against HIV-1 has already been assessed in MT-4 cells, and it was previously reported to have a half-maximal effective concentration (EC_50_) of 0.1 nM (11, 12). Because SHIV does not replicate in MT-4 cells, we performed an antiviral assay in primary rhesus PBMCs to measure anti-SHIV activity of LEN (Figure 2A). The resulting mean EC_50_ and EC_95_ values for LEN were 0.39 and 0.91 nM, respectively, against SHIV in rhesus PBMCs as compared with 0.05 and 0.12 nM, respectively, against a panel of 23 HIV-1 isolates of differing subtype, as previously reported for primary human PBMCs (11). After correcting for rhesus and human plasma protein binding, which informs the free drug concentration in plasma, LEN was predicted to be approximately 4.4-fold less potent against SHIV versus HIV in vivo (8.80 nM vs. 2.01 nM protein-adjusted EC_95_ [paEC_95_]; Table 2). The estimated LEN potency difference between SHIV in rhesus cells and HIV in human cells allowed us to compute the macaque model–adjusted clinical minimum target exposure as 70 nM (i.e., 4.4 × 16 nM human target trough concentration [C_trough_] exposure with 6-month dosing regimen).

Human plasma exposure to LEN with twice-yearly injections at 927 mg in a clinical formulation following 2 oral lead-in doses (on days 1 and 2) at 600 mg, has been previously described (13). In short, LEN concentrations reached an efficacious target of 15.5 ng/mL (or 16 nM) within 2 hours after injection and were maintained above this level through the dosing interval. Next, we characterized the pharmacokinetic profile of LEN preclinical formulation in macaques following a single subcutaneous administration of 5 dose levels, ranging from 5 to 75 mg per kilogram, in 4 animals per group. Animals dosed with 5–20 mg/kg LEN were monitored weekly for a minimum of 14 weeks, after which their exposures dipped below the paEC_95_ (8.8 nM) mark, while the 2 higher-dose groups were followed through study week 25. LEN had a slow, sustained release and dose-proportional increase in exposure from 5 to 20 mg/kg and a more than dose-proportional increase from 50 to 75 mg/kg in macaque plasma (Figure 2B and Supplemental Table 1). The slow release of LEN was demonstrated by a long half-life in the range of 17–53 days following a single subcutaneous dosing. LEN plasma drug levels peaked 4–17 days after administration on average for the 5, 10, 20, 50, and 75 mg/kg dosing groups.

### Safety and tolerability profile of subcutaneous LEN dosing in macaques.

Safety and tolerability of LEN was monitored via daily animal cage-side observations, injection site scoring according to the Draize grading scale daily for 2 weeks and then weekly through week 14 after dose, and hematology and clinical chemistry testing performed every other week for the first 4 weeks, followed by every 4 weeks through week 16. Injections at all dose levels were generally well tolerated without notable clinical observations. Grade 2–4 edema and erythema were noted at a single time point on week 1 after dosing among 2 animals in the 75 mg/kg group and 1 animal in the 50 mg/kg group. Grade 1 edema and/or erythema was observed across multiple time points and animals from all 5 dose groups and was fully resolved by week 11 after dose. Hematology analyses yielded no abnormal values throughout the study. Blood chemistry parameters remained within the normal range across all analytes, except for a transient minimal elevation in aspartate aminotransferase in 2 animals in the 75 and 5 mg/kg groups on weeks 2 and 4 after dosing, respectively. Overall, the subcutaneous injection of long-acting LEN was safe and well tolerated in rhesus macaques at all dose levels tested.

### SHIV infectivity in LEN-treated macaques.

**Figure 3:**
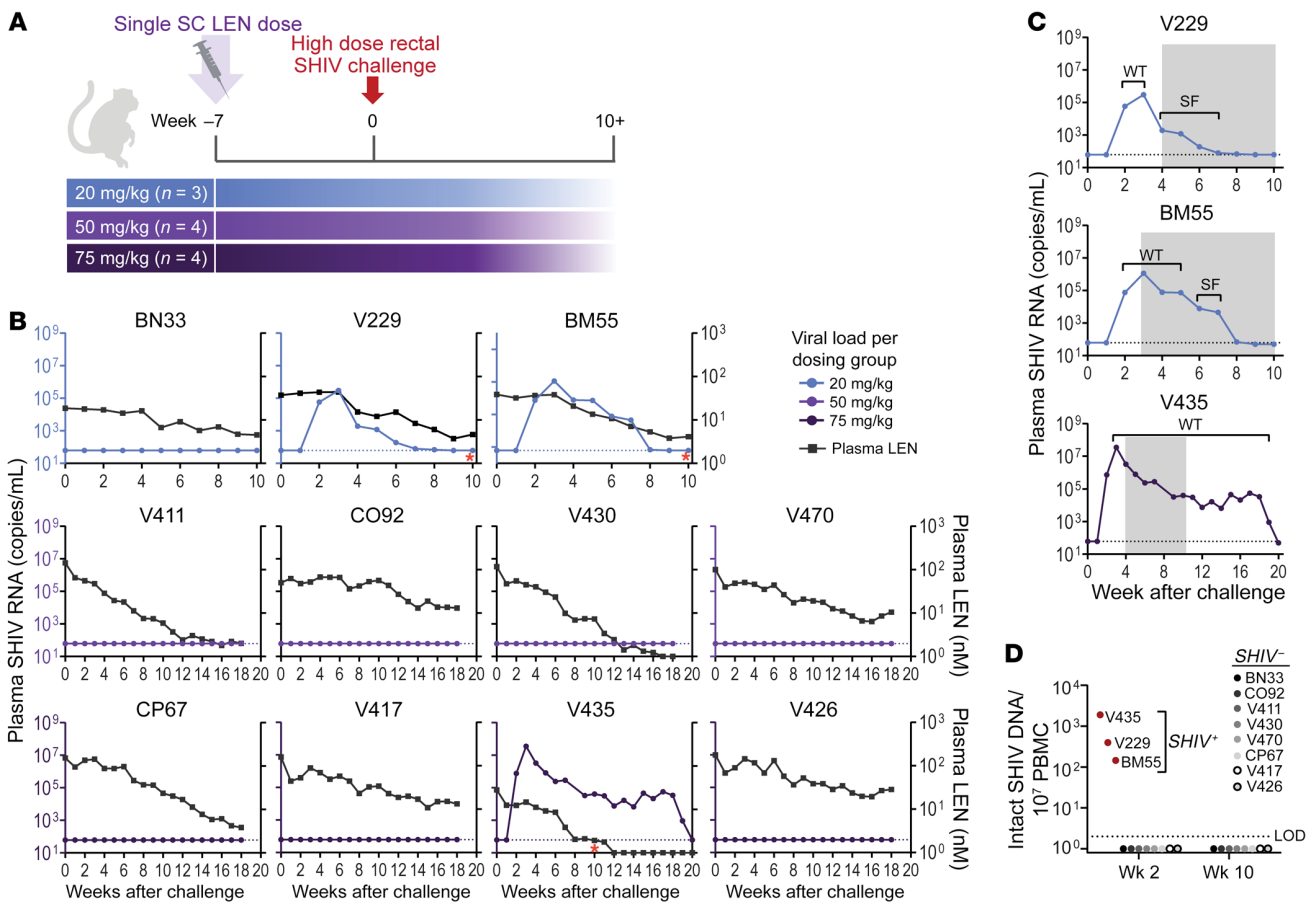
Study design, SHIV infectivity, seroconversion status, and capsid resistance profile after single challenge of LEN-treated macaques. (**A**) Study design. Rhesus macaques of Indian origin were treated with a single subcutaneous administration of LEN at the specified dose level on week 0 and challenged with a high-dose of SHIV on week 7. (**B**) Plasma SHIV viral loads measured by gag RT-qPCR among LEN-treated rhesus macaques after a single challenge with 100 TCID_50_ SHIV (plotted on the left *y* axis) versus plasma LEN exposure (plotted on the right *y* axis). Animals dosed with 20, 50, and 75 mg/kg LEN are represented by the top, middle, and bottom rows, respectively. Each symbol represents an average of 3 assay replicates. Dotted lines represent the assay limit of detection (LOD, 62 copies per mL). Asterisks indicate the timing of ART initiation among the 3 viremic animals. (**C**) The timing of p27 antibody detection (i.e., seroconversion) measured via serum ELISA and depicted by the gray shaded regions on corresponding plasma viral load curves (only animals with detectable signal shown of 11 assayed). Capsid-encoding gene sequencing results are noted above the viral load curves as applicable. WT, WT sequence; SF, sequence failure. (**D**) SHIV intact proviral DNA counts from PBMCs determined by intact proviral DNA assay among 11 SHIV-challenged rhesus macaques at the indicated time points. Each symbol represents an average of 12 technical replicates in a single experiment. Red symbols represent animals that were viremic by qPCR and seropositive by p27 serum ELISA. Dotted line represents the assay limit of detection (2 copies per million PBMCs).

To assess prophylactic efficacy of LEN a subset of 20 animals from the pharmacokinetic study was challenged rectally with SHIV on week 7 after LEN dosing. Based on “real-time” measurements through study week 5, LEN plasma exposures among 9 of 20 animals fell below 17.6 nM or 2 times paEC_95_ for LEN inhibition of SHIV, an index estimated to be protective with GS-CA1 in a prior repeat challenge study (10). Hence, 9 animals with insufficient LEN exposure were excluded from the efficacy assessment, while the remaining 11 animals from the groups receiving 20–75 mg/kg LEN doses received a single SHIV challenge (Figure 3A). The SHIV infection status in each challenged animal was initially established via weekly monitoring of plasma viral loads (Figure 3B). Of 11 animals, 3 became viremic 2 weeks after challenge (V229, BM55, and V435). Similar to the early SHIV replication kinetics observed among untreated animals in the virus titration study, viral loads peaked on week 3 and declined thereafter. Peak viremia was highly variable between animals, and no significant differences were observed between the untreated and LEN-dosed infected animals challenged with the same viral inoculum (Supplemental Figure 2). Between weeks 8 and 10 after infection, the 3 viremic animals were placed on ART to comply with a prespecified study criteria to prevent disease progression. Animals V229 and BM55 spontaneously controlled SHIV viremia ahead of ART initiation, likely owing to their expression of the major histocompatibility complex class I allele Mamu-B*17 implicated in the control of chronic-phase SIV replication (Supplemental Table 2) (14).

As a secondary measure of infection, we evaluated the seroconversion status of 11 SHIV-exposed animals using an anti-capsid (p27) antibody ELISA. All 3 animals positive for SHIV by qPCR produced measurable antibodies by week 4 after challenge, while the other 8 animals no antibodies were detectable through the end of the monitoring period on week 10 after challenge (Figure 3C). Third, we employed an intact proviral DNA assay (IPDA) to confirm the infection status and establish the timing of detectable reservoir establishment. We assayed PBMCs on weeks 1, 2, and 3 after challenge from the 3 animals with confirmed viremia and measured first detectable intact SHIV DNA coincidental with detectable viremia at week 2 after challenge (Figure 3D). Eight SHIV-negative animals by plasma PCR and serology were assayed by IPDA on weeks 2 and 10 to capture the early and late time points after challenge. No signal was detected at either time point, consistent with other assays, thus confirming uninfected status of 8 of 11 LEN-dosed animals challenged with SHIV.

We went on to characterize the viral capsid sequence from plasma-derived virus isolated from infected animals to look for any resistance emergence to LEN. Samples yielding sufficient SHIV RNA quantity and sequence data revealed no emerging mutations in capsid among the infected animals throughout the monitoring period (Figure 3C).

### LEN prophylactic efficacy relative to exposure.

**Figure 4:**
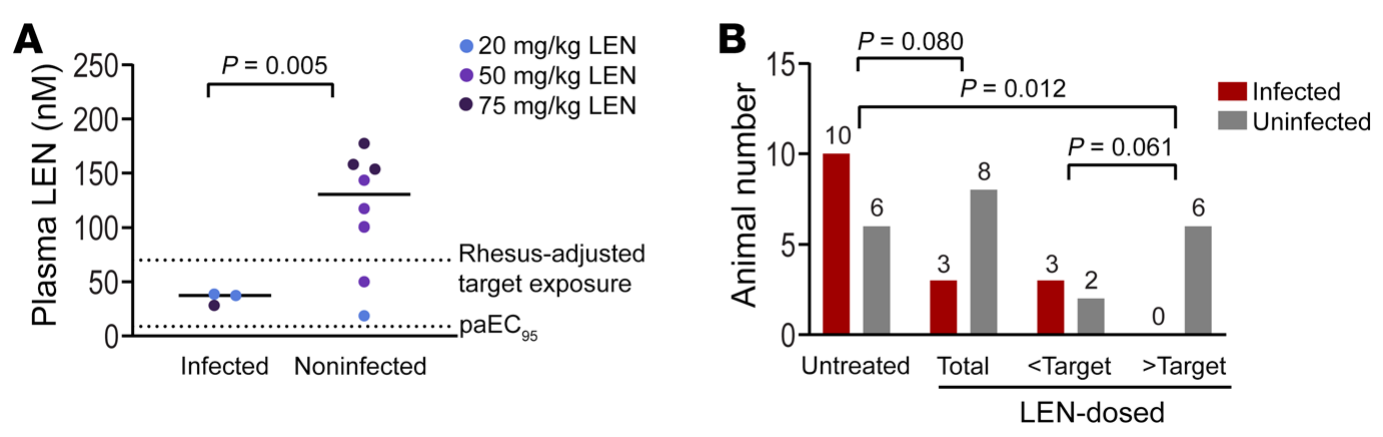
Animal infection status relative to LEN exposure at the time of challenge. (**A**) Plasma levels of LEN measured by mass spectrometry among SHIV-challenged animals at the time of challenge versus final infection status. Symbols represent plasma LEN concentration derived from an average of 3 biological assay replicates for individual study animals and are color-coded by the corresponding LEN-dosing groups. The horizontal bars represent group averages. The *P* value comparing exposures among infected versus uninfected animals, computed via an unpaired *t* test with Welch’s correction, is shown. The lower dotted line represents the rhesus paEC_95_ for LEN (8.8 nM), while the higher dotted line represents the rhesus-adjusted clinical target C_trough_ concentration for LEN (70 nM). (**B**) Infection rates among the different study animal subgroups. Bars represent the number of infected (red) or uninfected (gray) study animals that were not treated, the total number of animals dosed with LEN or dosed with LEN and split into subsets with exposures below or above the adjusted LEN target concentration. *P* values, computed via Fisher’s exact test, are shown.

To establish the precise LEN plasma levels at the time of challenge and compute LEN prophylactic efficacy, we plotted week 7 postdose drug exposure values for the infected and noninfected groups (Figure 4A). LEN exposures among 11 challenged animals ranged from 18 to 177 nM and were significantly lower in the infected group (*P* = 0.005, unpaired *t* test with Welch’s correction). To determine whether LEN offered significant protection against infection, we compared the rates of infection between total LEN-treated animals and untreated animals challenged with the same SHIV inoculum in the titration study. The infection percentage per exposure decreased from 62.5% among untreated animals (10 infections/16 challenges) to 27.2% (3 infections/11 challenges) among animals treated with LEN, though this infection rate reduction did not meet an α of 0.05 cutoff for significance (*P* = 0.08, 1-tailed Fisher’s exact test) (Figure 4B). However, considering that the LEN-treated group encompassed animals with exposures below the protective target, we performed a subgroup analysis based on the actual LEN exposure at the time of challenge. Hence, we evaluated protection above and below the model-adjusted clinical minimum target exposure of 70 nM calculated above. Above this target exposure cutoff, LEN provided complete protection against infection, with 0 of 6 animals becoming infected. When comparing the rate of infection in the untreated group to the LEN-treated group with exposures above the minimum target, LEN demonstrated significant protection against SHIV infection in this stringent nonhuman primate challenge model (*P* = 0.012, 1-tailed Fisher’s exact test).

## Discussion

Despite the high efficacy of currently available PrEP, new options are still needed, as only a minority of the people vulnerable to HIV infection are taking PrEP. Long-acting agents could address some of the reasons people have not been persistent in adherence to oral PrEP. Based on surveys, long-acting agents represent an acceptable and, in certain cases, preferred option for people who may benefit from PrEP (15). LEN, a first-in-class HIV capsid inhibitor suitable for twice-yearly subcutaneous administration, was recently approved for the treatment of multidrug-resistant HIV infection in combination with other antiretrovirals. Here, we examined the potential utility of LEN for HIV prophylaxis using a preclinical macaque SHIV challenge model.

We confirmed potent antiviral activity of LEN against SHIV in PBMCs, albeit with a slight increase in EC_50_ compared with HIV-1, as was the case for GS-CA1 (10). Amino acid variations within the capsid-encoding region between HIV-1 and SHIV, most notably at HIV-1 positions K70 and E180 (relative to the HXB2 reference strain) located within the LEN binding site, may account for lower potency against SHIV compared with HIV-1 (Supplemental Figure 3). Substitutions at both positions have been previously implicated in loss of susceptibility of HIV-1 to LEN in vitro and are also present in HIV-2, which is less susceptible to LEN (11, 16, 17).

We characterized the pharmacokinetic profile of a long-acting preclinical LEN formulation in rhesus macaques dosed with 5–75 mg/kg LEN, establishing a plasma drug half-life of 2–8 weeks in this species. Although we observed variability in key pharmacokinetic parameters (such as maximum concentration or area under the plasma concentration–time curve from time 0 to infinity across animals, in particular, in the 75 mg/kg dosing group due to animal V435 displaying a lower exposure profile), the overall group variance was consistent with that reported in the human clinical studies (13). Eleven animals belonging to the 3 highest LEN dosing groups maintained plasma exposures above the paEC_95_ or 8.8 nM for over 10 weeks and were chosen for the SHIV challenge study to establish LEN prophylactic efficacy. While a low-virus-dose repeat challenge model is useful in evaluating protection against multiple transmission events with a more physiologic virus inoculum, it can complicate accurate estimation of the time of infection and, hence, determination of the drug exposure that is expected to be protective. With only a single high-dose SHIV challenge, we interrogated protection with the precise knowledge of plasma drug exposure at the time of challenge. Compared with the untreated group, LEN administration reduced the rate of infection per exposure by more than 2-fold and was fully protective against SHIV acquisition at plasma drug exposures above the rhesus-adjusted C_trough_ concentrations targeted with twice-yearly formulations of LEN in the clinic. Unlike in the prior study with GS-CA1, peak viremia among LEN-dosed animals was not significantly inhibited relative to the control, which is likely a consequence of a smaller sample size in the current study (10).

A long-acting formulation of the integrase strand transfer inhibitor (INSTI) cabotegravir (CAB-LA) administered intramuscularly at 8-week intervals by a healthcare provider remains the only long-acting PrEP option approved to date. CAB-LA demonstrated superiority to the oral standard of care, attributed primarily to inadequate adherence to the daily tablet regimen (18). LEN, if demonstrated to be efficacious for PrEP, would have the potential to further transform the PrEP landscape with just twice-yearly dosing and simple subcutaneous administration.

In addition to LEN, various reverse transcriptase inhibitors, protease inhibitors, INSTIs, and entry inhibitors with long-acting potential are being evaluated as oral, injectable, and/or implantable agents for PrEP (19). Given that HIV prevention medicines are prescribed to an otherwise healthy population that may benefit from PrEP, a particularly high safety bar must be met to achieve a favorable risk-benefit ratio. Among the long-acting agents already in clinical development are broadly neutralizing antibodies (bNAbs), such as VRC01 to inhibit HIV entry, and the nucleoside reverse transcriptase translocation inhibitor islatravir to suppress virus replication. Although bNAbs are generally well tolerated, these biologics are limited in the breadth of viral strain coverage due to high diversity of HIV envelope sequence and are vulnerable to viral escape. VRC01 monotherapy did not prevent the overall HIV-1 acquisition but demonstrated reduced incidence of infection with a subset of sensitive strains (20). These data indicated that a combination of two or more bNAbs with broader strain coverage is likely needed to achieve clinical validation in future trials. Islatravir’s long half-life and high potency against HIV combined with its proven efficacy at preventing infection in a macaque model supported its evaluation for PrEP (21). However, unanticipated lymphopenia observed during clinical development resulted in a halt of ongoing PrEP studies with islatravir (22). Consistent with previously approved PrEP medicines, there have been no serious adverse events or laboratory abnormalities related to LEN reported through week 52 of treatment across clinical studies (4).

An additional important consideration for any PrEP medicine is the potential for selection of resistance in the case of breakthrough infections on PrEP, during the pharmacokinetic tail (i.e., long-term persistence of subprotective drug levels after the last dose) or when the initiation of PrEP coincides with an undetected HIV infection (23). Resistance to the first-line ART is of particular concern, as it would limit treatment options after infection (24). In the HPTN-083 study, evaluating CAB-LA for PrEP in cisgender men and transgender women who have sex with men, new infections and selection of INSTI-resistant variants were reported while on PrEP (25). Retrospective testing revealed that viral replication was suppressed for long periods by CAB-LA and that seroconversion, and hence HIV diagnosis, was significantly delayed (26). Results of phenotypic studies of emergent virus in these individuals confirmed resistance to CAB and, in some cases, other INSTI class drugs. Although follow up studies have suggested that earlier infection detection via an HIV RNA detection assay may help reduce the risk of resistance, the feasibility and merit of this approach remains to be addressed (27).

No resistance was selected among the 3 infections that occurred at suboptimal LEN exposures in the current study consistent with our previous macaque PrEP study conducted with the capsid inhibitor GS-CA1 (10). In addition, unlike with CAB-LA, none of the capsid mutations associated with resistance to LEN display cross-resistance to existing ART classes in vitro (11, 28). Resistance to LEN in HIV treatment clinical studies has been characterized, and it will be important to continue monitoring in ongoing trials for treatment and PrEP as well as in routine clinical practice (29, 30).

Together with our previous preclinical PrEP efficacy studies using the LEN analog GS-CA1, we established an important proof of concept that LEN can offer effective long-lasting HIV prophylaxis as a monotherapy. The measured half-life of LEN in macaques, though longer than that of GS-CA1, is still shorter than the 7–11 week range measured with the twice-yearly LEN formulation in human clinical studies (13, 31). Thus, human dosing is likely to provide longer-lasting protection against HIV infection in people than achievable in this macaque model. Two phase III clinical studies, PURPOSE 1 (NCT04994509) and PURPOSE 2 (NCT04925752), are currently underway to determine the safety and efficacy of subcutaneous long-acting LEN for PrEP among cisgender adolescent girls and young women in South Africa and Uganda and in cisgender men, transgender women, transgender men, and gender nonbinary individuals who have sex with men in North and South America, Africa, and Asia, respectively (32). Data presented in this manuscript further support the merit of evaluating LEN for PrEP in the clinic.

## Methods

### Drug and formulation.

LEN and the liquid chromatography–mass spectrometry internal standard GS-224337 were synthesized at Gilead Sciences Inc. and subjected to a standard quality control analysis. For antiviral assays, LEN was dissolved in DMSO to produce a 10 mM stock concentration and stored frozen at –0°C. For animal dosing studies, LEN was dissolved in vehicle (58.03% polyethylene glycol 300, 27.1% water, 6.78% ethanol, 6.61% poloxamer 188, 1.48% sodium hydroxide) at 300 mg/mL, stored at ambient temperature, and protected from light until dosing. The formulation contained additional excipients absent from the clinical formulation in order to tailor the pharmacokinetic profile in macaques.

### Virus.

The SHIV-162P3 virus was derived from SIV_mac239_ and encodes the HIV genes env, tat, rev, and vpu. The molecular clone was generated through the intravenous infusion and infection of rhesus macaques followed by 3 consecutive serial blood/bone marrow transfusions. The parent stock was obtained from the laboratory of Dan Barouch at Beth Israel Deaconess Medical Center, Harvard Medical School (Boston, Massachusetts, USA) and in vitro expanded in concanavalin A–activated PBMCs from specific pathogen–free rhesus macaques. In vitro titration was performed in phytohemagglutinin-stimulated (PHA-stimulated) rhesus PBMCs infected with a serial dilutional series of viral harvest. The cultures were tested for the presence of SIV capsid (p27) in the supernatant using an antigen capture assay (ABL Inc.). Wells that showed an assay OD value greater than the virus alone control wells were considered positive. The number of SIV-positive versus -negative wells at each serial dilution was enumerated and the TCID_50_ was calculated.

### Primary cells.

Freshly isolated PBMCs from 3 male Indian rhesus macaque (Macaca mulatta) donors were obtained from Human Cells Biosciences. Rhesus PBMCs were cultured in RPMI-1640 cell culture media (Life Technologies) supplemented with 10% heat-inactivated FBS (Hyclone), 2 mM glutamine, and 100 units/mL penicillin plus 100 μg/mL streptomycin (complete RPMI). Prior to their use in antiviral assays, PBMCs from 3 independent donors were pooled and activated at a density of 3 × 10^6^ cells per mL for 72 hours at 37°C by addition of 1 μg/mL PHA (Sigma-Aldrich) and 50 units/mL recombinant human IL-2 (Roche Diagnostics).

### Antiviral assay in PBMCs.

Antiviral assays for LEN in rhesus PBMCs acutely infected with SHIV.SF162P3 were performed as previously described for HIV-1 in human PBMCs, with minor modifications (11). Briefly, PHA/IL-2–stimulated rhesus PBMCs were infected in bulk culture with SHIV.SF162P3 at a concentration of 130 pg p27 equivalent per million PBMCs. Cells were maintained in suspension by gently rocking the cultures mixed with virus inoculum for 3 hours at 37°C. Cells were then pelleted by centrifugation at 500*g* for 5 minutes, washed twice with complete RPMI to remove any unadsorbed virus, and seeded into 96-well plates at a cell density of 2 × 10^5^ cells per well in 100 μL. Eight-point 3-fold serial dilutions of LEN were made in complete RPMI containing 50 units/mL IL-2 and added in triplicate to wells containing cells (100 μL per well). Cultures were incubated in a 5% CO_2_ incubator at 37°C for 7 days. The cell-free supernatants derived from the PBMC cultures were harvested 7 days after infection, and the amount of SHIV present was quantified using a SIV p27 antigen capture ELISA assay (item 5436, Advanced Bioscience Laboratories Inc.) performed according to the manufacturer’s protocol. The mean EC_50_ value for LEN, determined from a total of 7 assays across 2 independent experiments, was calculated from the dose-response curves using XLfit software (IDBS). The Hill coefficient (*n*) for LEN was measured from the slope of antiviral dose response curves (*n* = 3.51 ± 0.31) and used to calculate its EC_95_ value using the following equation: EC_95_ = EC_50_ × (95/5)^1/*n*^.

### Equilibrium dialysis shift assay.

Rhesus plasma protein binding to LEN was determined by competitive equilibrium dialysis. Pooled rhesus plasma (10%) from 12 animals was spiked with LEN (2 μM), and this mixture and blank RPMI cell culture medium containing 10% FBS (cell culture medium [CCM]) were placed into opposite sides of assembled dialysis cells. The incubations were performed in triplicate. After a 24-hour equilibration period at 37°C, samples were collected and mixed with opposite side of blank matrix. Samples were deproteinated with 4-volume equivalents of acetonitrile containing internal standard, and the resulting supernatant was analyzed by liquid chromatography–mass spectrometry/MS. LEN and the internal standard were detected using a source that was configured with Turbo Ion Spray ionization in positive mode and using multiple-reaction monitoring. The fold change value in 100% rhesus plasma was then calculated using the plasma/CCM ratio after correcting for the sample dilution factor and the percentage-free fraction in the matrix.

### Animal studies.

All animals were housed at Bioqual Inc. For the in vivo SHIV stock titration study, 8 untreated outbred Indian-origin male rhesus macaques aged 3–5 years were challenged intrarectally per round for a total of 5 challenge rounds using increasing virus doses ranging from 0.625 to 100 TCID_50_, with the 100 TCID_50_ round performed twice for increased resolution (Supplemental Table 2). Plasma viral load was measured to confirm the infection status. For LEN pharmacokinetics and PrEP efficacy determination, 20 outbred Indian-origin male rhesus macaques aged 3–5 years were assigned to 5 study groups with an even weight distribution (Supplemental Table 2). On study week 0, 4 animals per group were administered LEN at 5, 10, 20, 50, or 75 mg/kg in the scapular region by subcutaneous injection. LEN was prepared as a 300 mg/mL stock solution, and no more than 2 mL solution was injected into a single subcutaneous site. Injection sites were monitored daily by veterinary staff for 2 weeks and then weekly through the end of study. On week 7, 11 animals were challenged by the intrarectal route with 1 mL RPMI containing 100 TCID_50_ SHIV-SF162P3. Whole blood was collected and processed into plasma and PBMCs as necessary for the assessment of routine hematology and clinical chemistry, viral load analysis, serology, and the bioanalysis of drug levels. Animals were considered protected if they remained SHIV negative by a plasma PCR assay and seronegative by enzyme immunoassay through week 10 after challenge.

Animals confirmed as SHIV positive in both the virus titration and the PrEP efficacy studies were placed on a daily subcutaneous ART regimen between weeks 4 and 10 after infection to prevent AIDS disease progression. The formulated ART cocktail (Gilead Sciences Inc.) contained tenofovir disoproxil fumarate (5.1 mg/mL), emtricitabine (40 mg/mL), and dolutegravir (2.5 mg/mL) and was administered subcutaneously once daily at 1 mL/kg.

### Bioanalysis of LEN in macaque plasma.

Rhesus plasma samples were stored frozen at –80°C and thawed, and a 50 μL aliquot of each was treated with 200 μL acetonitrile containing an internal standard. After precipitation of the protein component, a 100 μL aliquot of the supernatant was transferred to a clean 96-well plate and mixed with 200 μL water. A 10 μL aliquot of the above solution was then injected into a Q-Exactive high resolution mass spectrometer from Thermo Electron with electrospray ionization in positive mode. Quantification was performed using an accurate mass ([M+H]+) of 968.1508 for LEN and 756.3289 for the internal standard. The lower and upper limits of quantitation for LEN were 1 nM and 10,000 nM, respectively.

### Plasma viral load assay.

A QIAsymphony SP (Qiagen) automated sample preparation platform along with a Virus/Pathogen DSP midi kit and the cellfree500 protocol were used to extract viral RNA from 500 μL plasma. A reverse primer specific to the gag gene of SIVmac251 (5′-CACTAGGTGTCTCTGCACTATCTGTTTTG-3′) was annealed to the extracted RNA and then reverse transcribed into cDNA using SuperScript III Reverse Transcriptase (Thermo Fisher Scientific) along with RNAse Out (Thermo Fisher Scientific). The resulting cDNA was treated with RNase H (Thermo Fisher Scientific) and then added (2 replicates) to a custom 4× TaqMan Gene Expression Master Mix (Thermo Fisher Scientific) containing primers and a fluorescently labeled hydrolysis probe specific for the gag gene of SIVmac251 (forward primer 5′-GTCTGCGTCATCTGGTGCATTC-3′, reverse primer 5′-CACTAGGTGTCTCTGCACTATCTGTTTTG-3′, probe 5′-/56-FAM/CTTCCTCAGTGTGTTTCACTTTCTCTTCTGCG/3BHQ_1/-3′). The qPCR was then carried out on a QuantStudio 3 Real-Time PCR System (Thermo Fisher Scientific). Mean SIV gag RNA copies per reaction were interpolated using quantification cycle data and a serial dilution of a highly characterized custom RNA transcript containing a 730 bp sequence of the SIV gag gene. The assay limit of quantification is approximately 62 RNA copies per milliliter of sample.

### ELISA.

Rhesus serum samples from viremic study animals were tested for the presence of antibodies against HIV-1 by ELISA using the GS HIV-1/HIV-2 PLUS O EIA assay kit from Bio-Rad (catalog 32588). Individual macaque sera (150 μL) mixed with 50 μL specimen diluent supplied in the kit were added to assay plates precoated with recombinant purified HIV-1 capsid (p24) protein and transmembrane glycoprotein (gp160) and incubated for 1 hour at room temperature. The plates were then washed 3 times with a sodium chloride and Tween 20–containing wash buffer from the kit and incubated for 1 hour with a HRP-conjugated antigen solution containing peptides mimicking various immunodominant epitopes of HIV-1 gp160 and p24 proteins. Wells with antibody against HIV-1 bound to the antigen coating the wells and to the peroxidase-conjugated antigens in the conjugate solution to form immobilized stable antigen-antibody-antigen complexes. The plates were washed 3 more times with the above wash buffer, developed with a working solution of tetramethylbenzidine, stopped by the addition of 1 N sulfuric acid, and analyzed at 450 nm using a Versamax microplate reader (Molecular Devices) using the Softmax Pro 6.5.1 software. Samples with an OD450 nm absorbance value of more than 0.2 were considered positive.

### IPDA.

The SHIV-adapted version of IPDA (SHIV-IPDA) was used to determine the number of intact SHIV proviruses. Total genomic DNA was extracted from unfractionated PBMCs using a QIAamp DNA Mini kit (Qiagen). DNA quality and quantity were evaluated by spectrophotometry and fluorometry, respectively, and SHIV-IPDA was then performed on the isolated DNA. In brief, SHIV-IPDA consists of a 3-component multiplex droplet-digital PCR (ddPCR) reaction. The first is a SHIV proviral discrimination reaction targeting two conserved, frequently deleted regions of the SHIV genome to determine the intact provirus count; the second is a 2-long terminal repeat (2-LTR) DNA circle reaction to determine 2-LTR circle counts; and the third is a copy reference/DNA-shearing reaction targeting ribonuclease P/MRP subunit P30 (RPP30) to determine assay input cell equivalents and the DNA shearing index. All ddPCR reactions were performed using a Bio-Rad QX200 AutoDG ddPCR system with Bio-Rad ddPCR supermix for probes with no dUTP. After DNA shearing index correction and subtraction of intact 2-LTR circles, the intact proviral frequencies were reported per million input cells. The endpoint ddPCR data were collected using Bio-Rad QuantaSoft version 1.7.4.0917.

### Plasma virus genotypic analysis.

Total RNA was extracted from 50 μL plasma aliquots obtained from each viremic monkey using the MagMAX-96 Viral RNA Isolation Kit (Life Technologies) in conjunction with the Thermo Fisher Scientific KingFisher Flex automated extraction platform and eluted in 60 μL AVE buffer. The capsid coding area of *gag* in each sample was then individually amplified by RT-PCR using the SuperScript IV One-Step RT-PCR System (Life Technologies) and the Qiagen OneStep RT-PCR Kit according to the manufacturers’ recommended protocols. Amplification of the SHIV capsid coding region in each sample was performed using primers (SIV-CA-F [5′-CCAAAAACAAGTAGACCAACAG-3′] and SIV-CA-R [5′-TGCAAAAGGGATTGGCAC-3′]) and the products subjected to population-level bulk sequencing at Elim Biopharmaceuticals Inc. using the same primer set. To identify codon changes, capsid encoding sequences for each sample were aligned using DNA Sequencher Software (Gene Codes Corporation) with that of the parent challenge virus stock. A sequence alignment for major consensus HIV-1 subtype, HIV-2, and SHIV-SF162P3 capsid amino acid sequences was performed using BioEdit Sequence Alignment Editor version 7.2.6.

### Statistics.

Protection against acquisition of infection was analyzed using Fisher’s exact test. Comparisons were considered statistically significant at a 2-sided α level of 0.05 (*P* < 0.05). Animal infectious dose modeling was done based on logistic regression represented by the following formula: 

(Equation 1)


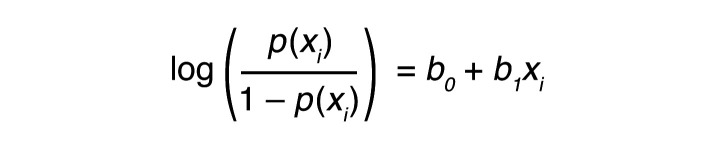


where *p*(*x*) represents the infection rate at dose *x*_*i*, with *i* representing doses 1, 2, and 3. The response is binary with responder represented as 1 and nonresponder represented as 0. The parameter estimates were obtained from the dose-response curve package in R.

### Study approval.

All animal procedures were conducted in compliance with the relevant local, state, and federal regulations and were approved by the Bioqual Inc. IACUC (Rockville, Maryland, USA). Human PBMCs were collected from healthy volunteers after informed consent, and their use was approved by an institutional review board at AllCells (Alameda, California, USA).

### Data availability.

Values for all data points in graphs are reported in the Supporting Data Values file.

## Author contributions

EB designed and oversaw the animal virus titration study. EB, SRY, and CC designed the pharmacokinetic and efficacy animal studies. EB and LV oversaw the pharmacokinetic and efficacy study execution. DH performed and analyzed the antiviral and serology assays and viral sequencing. KW performed and analyzed the equilibrium dialysis shift assays. WR supported drug formulation. BL and SRY analyzed the plasma LEN concentrations. EB interpreted the data and wrote the manuscript with input from all authors.

## Supplementary Material

Supplemental data

Supporting data values

## Figures and Tables

**Table 2:**
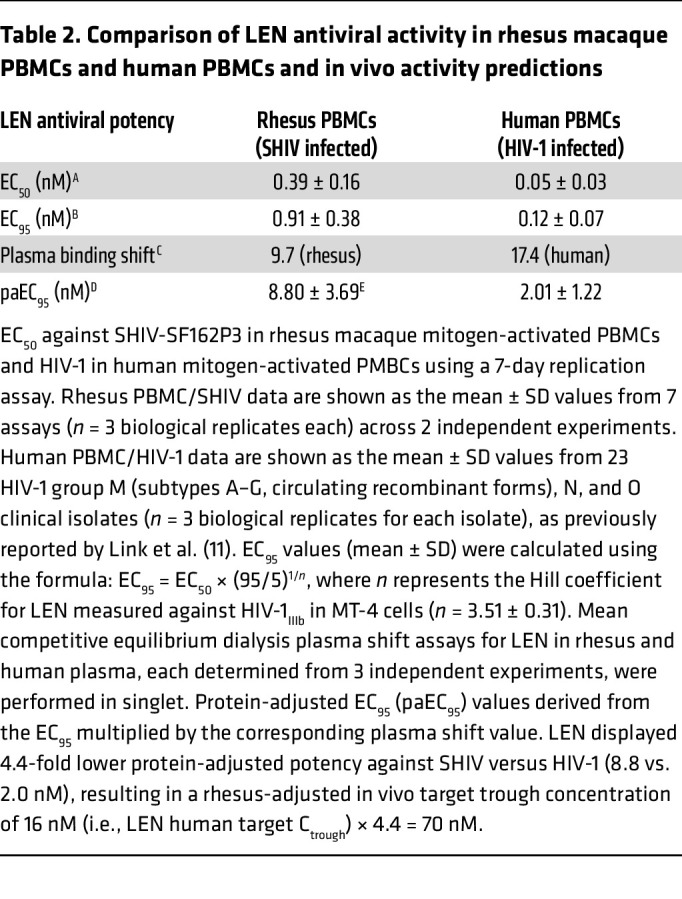
Comparison of LEN antiviral activity in rhesus macaque PBMCs and human PBMCs and in vivo activity predictions

**Table 1:**
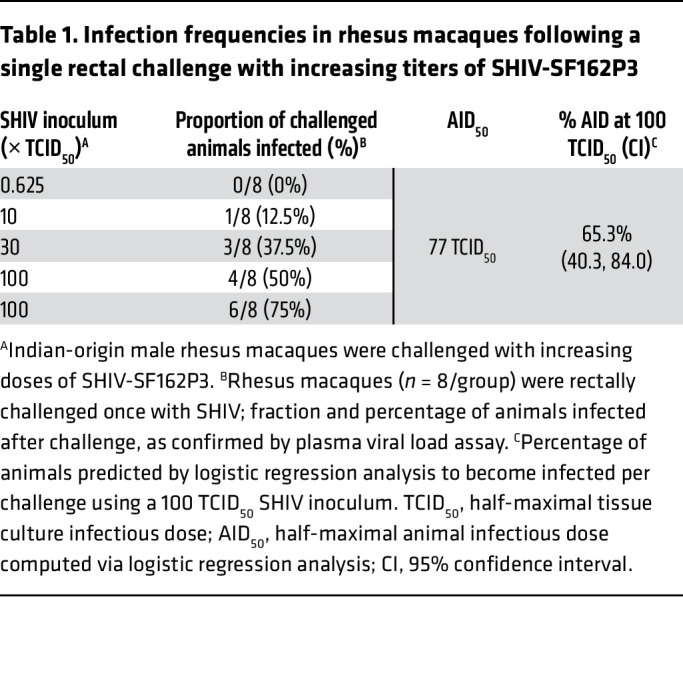
Infection frequencies in rhesus macaques following a single rectal challenge with increasing titers of SHIV-SF162P3

## Version 1 06/29/2023

In-Press Preview

## Version 2 08/15/2023

Electronic publication
