# Supplementary material for: Long-acting lenacapavir acts as an effective preexposure prophylaxis in a rectal SHIV challenge macaque model
Source: J Clin Invest. 2023 Aug 15;133(16):e167818. doi: 10.1172/JCI167818 (PMC10425210; doi:10.1172/JCI167818)
Supplement: Supplemental data [file jci-133-167818-s037.pdf]

Supplemental material

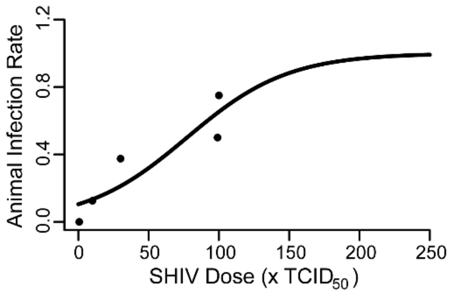

Supplemental Figure 1. SHIV animal infectious dose modeling via logistic regression. Curve fit using logistic regression modeling for SHIV doses expressed in multiples of $TCID_{50}$ vs animal infection rate. The projected half-maximal animal infectious dose (AID_{50}) for this SHIV-SF162P3 stock was 77 $TCID_{50}$.

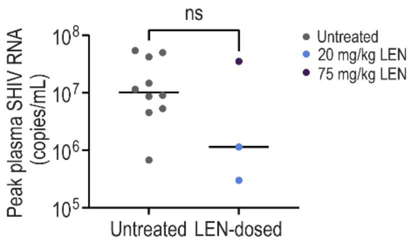

Supplemental Figure 2. Peak viremia. Peak viremia as measured by weekly plasma SHIV RNA PCR. Symbols represent individual animals color-coded by LEN-dosing group or untreated. Statistical comparison conducted via Fisher's exact test (ns, not significant).

```
HIV-1_CON_B     40  F SALSEGATPQ DLNTMLNTVG GHQAAMQMLK ETINEEAAEW DRLHPVHA
HIV-1_CON_A1    40  . .......... ...M...I.. .......... D......... ........
HIV-1_CON_A2    40  . T......... .......... .......... D......... ........
HIV-1_CON_C     40  . T......... .......... .......... D......... ........
HIV-1_CON_D     40  . .......... .......... .......... .......... ........
HIV-1_CON_F1    40  . .......... .......... .......... D......... ........
HIV-1_CON_G     40  . .......... .......... .......... D......... ..M..QQ.
HIV-1_CON_H     40  . .......... ...A...... .......... D......... ........
HIV-1_CON_01    40  . .......... ...M...I.. .......... .......... ..V.....
HIV-1_CON_02    40  . .......... ...M...I.. .......... D......... ..V.....
HIV-2_NIHZ      40  . Q.....C..Y .I.Q...C.. D......IIR .I......D. .VA..IP~
SHIV_SF162P3    40  . Q.....C..Y .I.Q...C.. D......IIR DI......D. .LQ..QP.

HIV-1_CON_B     90  P IAPGQMREPR GSDIAGTTST LQEQIGWMTN NP~PIPVGEI YKRWIILG
HIV-1_CON_A1    90  . .P........ .......... P........G ..~.....D. ........
HIV-1_CON_A2    90  . .P........ .......... .........S ..~....... ........
HIV-1_CON_C     90  . .......... .......... .....A...S ..~.....D. ........
HIV-1_CON_D     90  . V......... .......... .........S ..~....... ........
HIV-1_CON_F1    90  . .P........ .......... .....Q...S ..~.V...D. ........
HIV-1_CON_G     90  . .P...I.... .......... .....R...S ..~....... ........
HIV-1_CON_H     90  . .P........ .......... .....A...G ..~.....D. ........
HIV-1_CON_01    90  . .P........ .......... .......... ..~.....D. ........
HIV-1_CON_02    90  . .P........ .......... .........S ..~....... .....V..
HIV-2_NIHZ      89  . LPA..L.... .......... VE...Q..FR PQN.V...N. .R...QI.
SHIV_SF162P3    89  . ~QQ..L...S .........S VD...Q..YR QQN.....N. .R...Q..

HIV-1_CON_B    139  N KIVRMYSPTS ILDIRQGPKE PFRDYVDRFY KTLRAEQASQ E 180
HIV-1_CON_A1   139  . ........V. ....K..... .........F ........T. . 180
HIV-1_CON_A2   139  . ........V. .......... .........F ........T. . 180
HIV-1_CON_C    139  . ........V. ....K..... .........F ........T. D 180
HIV-1_CON_D    139  . ........V. .......... .......... .......... D 180
HIV-1_CON_F1   139  . ........V. .......... .........F ........T. . 180
HIV-1_CON_G    139  . ........V. .......... .........F ........T. . 180
HIV-1_CON_H    139  . ........V. ....K..... .........F ........T. D 180
HIV-1_CON_01   139  . ........V. .......... .......... ........T. . 180
HIV-1_CON_02   139  . ........V. .......... .........F ........T. . 180
HIV-2_NIHZ     139  Q .C....N..N ....N..... ..QS...... .S.....TDP A 180
SHIV_SF162P3   138  Q .C....N..N ...VK..... ..QS...... .S.....TDA A 179
```

Supplemental Figure 3. Alignment of HIV-1 (subtype consensuses), HIV-2 (NIHZ strain) and SHIV-SF162P3 capsid amino acid sequences. Dots represent invariant residues within the LEN binding site contained within residues 40-180 of HIV-1 capsid, tilde symbols denote sequence gaps, and red arrows highlight HIV-1 residues associated, either alone or in combination, with reduced in vitro susceptibility to LEN (L56, N57, M66, Q67, K70, N74, A105, T107, E180). Boxed sequences highlight the substitutions in HIV-2 and SHIV capsids most likely responsible for reduced susceptibility to LEN.

Supplemental Table 1. Pharmacokinetic parameters for rhesus macaques dosed with subcutaneous LEN

	5 mg/kg		10 mg/kg		20 mg/kg		50 mg/kg		75 mg/kg	
	Mean ± SD	CV%	Mean ± SD	CV%	Mean ± SD	CV%	Mean ± SD	CV%	Mean ± SD	CV%
**AUC_{last} (µM•h)**	9.3 ± 1.9	21	20.3 ± 1.7	8	48.4 ± 17.9	37	179 ± 23	13	284 ± 126	44
**AUC_{inf} (µM•h)**	10.6 ± 1.7	16	23.8 ± 0.6	2	53.0 ± 16.7	32	243 ± 70	29	367 ± 239	65
**$t_{1/2}$ (h)**	17.2 ± 5.0	29	25.1 ± 9.1	36	18.7 ± 7.1	35	45.1 ± 19.8	44	53.1 ± 39.1	74
**C_{max} (nM)**	10.5 ± 5.4	51	21.5 ± 2.0	9	42.5 ± 14.2	33	200 ± 74	37	530 ± 241	45
**T_{max} (h)**	408 ± 736	180	240 ± 400	167	228 ± 408	179	102 ± 156	153	102 ± 156	153

Analytes include area under the plasma concentration–time curve from time 0 to the last quantifiable time point (AUC_{last}), area under the plasma concentration–time curve from time 0 to infinity (AUC_{inf}), maximal concentration (C_{max}), time to reach observed peak plasma concentration (T_{max}), and terminal half-life ($t_{1/2}$) determined by non-compartmental analysis using Phoenix WinNonlin 6.4 build 8.1.0.3530. CV, coefficient of variation (100 X standard deviation / mean). [a]Percent of area under the plasma concentration–time curve extrapolated between AUC_{inf} and AUC_{last} ranged from 8.7% to 26.3%.

Supplemental Table 2. Study animal details

Study group	Animal ID	Sex	Age (years)	Weight (kg)	Mamu controller allele	SHIV Rectal Challenge	Challenge outcome
0.625 $TCID_{50}$ SHIV titration	40941	male	10.5	11.3	A*01+	yes	Not infected
	43689	male	7.5	9.6	Triple negative	yes	Not infected
	44519	male	6.5	9.0	A*01+	yes	Not infected
	44632	male	6.5	11.8	Triple negative	yes	Not infected
	150795	male	5.6	8.4	Triple negative	yes	Not infected
	V234	male	5.5	6.8	Triple negative	yes	Not infected
	V227	male	5.4	6.4	B*17+	yes	Not infected
	V236	male	5.2	7.9	B*08+	yes	Not infected
10 $TCID_{50}$ SHIV titration	40941	male	10.6	11.3	A*01+	yes	Infected
	43689	male	7.6	9.6	Triple negative	yes	Not infected
	44519	male	6.6	9.0	A*01+	yes	Not infected
	44632	male	6.5	11.8	Triple negative	yes	Not infected
	150795	male	5.7	8.4	Triple negative	yes	Not infected
	V234	male	5.6	6.8	Triple negative	yes	Not infected
	V227	male	5.5	6.4	B*17+	yes	Not infected
	V236	male	5.3	7.9	B*08+	yes	Not infected
100 $TCID_{50}$ SHIV titration	43689	male	7.6	9.6	Triple negative	yes	Infected
	44519	male	6.6	9.0	A*01+	yes	Not infected
	44632	male	6.6	11.8	Triple negative	yes	Infected
	150795	male	5.7	8.4	Triple negative	yes	Infected
	V234	male	5.7	6.8	Triple negative	yes	Infected
	V227	male	5.5	6.4	B*17+	yes	Infected
	V236	male	5.4	7.9	B*08+	yes	Not infected
	35496	male	4.6	5.7	Triple negative	yes	Infected
30 $TCID_{50}$ SHIV titration	44519	male	6.7	9.0	A*01+	yes	Infected
	V236	male	5.4	7.9	B*08+	yes	Not infected
	V232	male	2.8	6.6	B*17+	yes	Not infected
	CL09	male	4.8	4.5	Triple negative	yes	Not infected
	MA313	male	4.8	4.9	A*01+	yes	Infected
	CL27	male	4.7	5.0	Triple negative	yes	Not infected
	41066	male	10.7	11.0	Triple negative	yes	Infected
	CS14	male	4.5	4.5	Triple negative	yes	Not infected
100 $TCID_{50}$ SHIV titration (round 2)	CV32	male	3.9	4.2	Triple negative	yes	Not infected
	BM71	male	3.9	4.3	Triple negative	yes	Infected
	CL51	male	3.9	5	Triple negative	yes	Infected
	V236	male	5.5	7.9	B*08+	yes	Not infected
	V232	male	2.8	6.6	B*17+	yes	Infected
	CS14	male	4.5	4.5	Triple negative	yes	Not infected
	CL09	male	4.9	4.5	Triple negative	yes	Infected
	CL27	male	4.7	5.0	Triple negative	yes	Not infected
5 mg/kg LEN PK	CV32	Male	4.2	7.3	Triple negative	no	n/a
	BM13	Male	5.1	6.9	A*01+	no	n/a
	V236	Male	5.8	10.8	B*08+	no	n/a
	BN09	Male	3.8	6.5	B*17+	no	n/a
10 mg/kg LEN PK	CS14	Male	4.9	7.8	Triple negative	no	n/a
	BM85	Male	4.1	6.4	Triple negative	no	n/a
	V233	Male	4.1	6.2	Triple negative	no	n/a
	BN13	Male	3.8	6.4	Triple negative	no	n/a
20 mg/kg LEN PK / efficacy	CL27	Male	5.1	7.8	Triple negative	no	n/a
	BN33	Male	3.5	4.6	Triple negative	yes	Not infected
	V229	Male	3.4	5.3	B*17+	yes	Infected
	BM55	Male	4.6	6.4	B*17+	yes	Infected
50 mg/kg LEN PK / efficacy	V411	Male	2.9	4.4	Triple negative	yes	Not infected
	CO92	Male	5.2	7.5	Triple negative	yes	Not infected
	V430	Male	2.9	4.0	A*01+	yes	Not infected
	V470	Male	2.4	3.2	A*01+	yes	Not infected
75 mg/kg LEN PK / efficacy	CP67	Male	4.4	7.8	Triple negative	yes	Not infected
	V417	Male	2.6	3.4	Triple negative	yes	Not infected
	V435	Male	3.1	4.1	A*01+	yes	Infected
	V426	Male	3.1	4.2	A*01+	yes	Not infected

Indian origin rhesus macaques used in the SHIV titration, PK and/or PrEP efficacy studies. Animal ID, sex, age, weight, major histocompatibility complex class I controller-allele status (based on typing results for Mamu*A01, *B08 and *B17 controller alleles), exposure to SHIV and SHIV challenge outcome as applicable are listed.
